# Comparison of intestinal and environmental microbiota of the snapping shrimp (*Alpheus brevicristatus*) in a seagrass bed

**DOI:** 10.3389/fmicb.2025.1735708

**Published:** 2026-01-09

**Authors:** Fang-Chao Zhu, Yan-Bin Yang, Qun-Jian Yin, Xu-Yang Chen, Shuo Yu

**Affiliations:** 1 Guangxi Laboratory of Oceanography, Beihai, China; 2 Key Laboratory of Tropical Marine Ecosystem and Bioresource, Fourth Institute of Oceanography, Ministry of Natural Resources, Beihai, China; 3 Guangxi Key Laboratory of Beibu Gulf Marine Resources, Environment and Sustainable Development, Fourth Institute of Oceanography, Ministry of Natural Resources, Beihai, China; 4 Observation and Research Station of Coastal Wetland Ecosystem in Beibu Gulf, Ministry of Natural Resources, Beihai, China; 5 School of Resources, Environment and Materials, Guangxi University, Nanning, China

**Keywords:** assembly mechanism, bacterial community, environment, intestine, snapping shrimp

## Abstract

Symbiotic bacteria associated with benthic invertebrates in seagrass beds play an important role in mediating host adaptability and maintaining ecosystem health; however, the taxonomic composition and functional characteristics of the symbiotic microbiota in these invertebrates remain poorly understood. In this study, the intestinal microbiota of seagrass bed-associated snapping shrimp *Alpheus brevicristatus* was characterized, and their composition was further compared with that of surrounding seawater and sediment using 16S amplicon sequencing. Our results revealed that the intestinal microbiota were dominated by unclassified Alphaproteobacteria and *Vibrio*. Compared to that of the environment, the microbiota of shrimp intestines showed lower alpha diversity, yet distinct microbial assemblages. Shrimp intestinal microbiota shared more species with sediment than seawater microbiota, suggesting sediment as a primary microbial source. Beta diversity analysis showed marked differences in microbial structure among habitats. The neutral community model and null model analyses indicated that stochastic processes exerted a significant influence on intestinal microbiome assembly. These findings highlight the complex interplay between host physiology and environmental exposure in shaping intestinal microbiota, providing foundational insights into host-microbe-environment interactions in benthic marine invertebrates.

## 1 Introduction

Intestinal microbiota refers to the diverse community of microorganisms, including bacteria, viruses and protists, that reside in the digestive tract of host. These microorganisms often act as mutualistic symbionts and play crucial roles in multiple host functions, such as development, nutrition, pathogen resistance, and immune regulation (Adak and Khan, 2019; Shamjana et al., 2024). However, our current knowledge about intestinal microbiota and its benefits are mainly obtained from studies on vertebrates and arthropods, particularly on mammals and insects. The intestinal microbiota of most invertebrates is still poorly understood. Invertebrates represent the largest group of animals on earth, comprising nearly 95% of all species within the animal kingdom (Eisenhauer and Hines, 2021). The digestive systems of invertebrates harbor a wide variety of evolutionarily diverse microbes, and have developed virtually every known type of beneficial host–microbe interaction (Petersen and Osvatic, 2018). So, it’s difficult to identify important symbiotic bacteria and to understand the mechanism of microbial community assembly.

Host-associated traits, such as developmental stage and genetic background, are primary determinants of intestinal microbial composition across diverse animal species (Gao et al., 2023). The host evolutionary history usually determines the “basic framework” of its intestinal microbiota, as evidenced by phylosymbiosis, a pattern where host phylogenetic relationships correlate with intestinal microbial similarity in insects and decapod shrimps (Tang et al., 2021; Qin et al., 2023). Beyond host factors, neutral models and environmental factors (e.g., diet, biogeography, pollutants, drugs) can effectively explain the intestinal microbiome composition in animals (Maritan et al., 2024). A typical example is termites which feed on lignocellulose and rely on their gut microbiota to break down the plant fibers (Brune, 2014). In aquatic animals, the microbial community thriving in the host’s intestine ecosystem is determined by the surrounding environment, primarily through ingested food material, sediment and water (Sun et al., 2019). Generally, the prevailing view holds that microbial community assembly is driven by both deterministic and stochastic processes (Yuan et al., 2019). Deterministic processes include environmental filtering, abiotic selection, and biological interactions, while stochastic processes (also known as neutral processes) include dispersal-related processes and ecological drift (Flores et al., 2025). Despite extensive sampling efforts, the relative contributions of these ecological processes and the mechanisms by which they collectively shape variations in host-associated microbial communities across different animals remain unclear.

Snapping shrimp (family Alpheidae) exhibits an extraordinary level of species diversity, comprising more than 700 recognized species distributed across 48 established genera (Sha et al., 2019). They typically burrow in soft sediment or live under rocks, shells and coral reefs, and are a ubiquitous component of macrofaunal assemblages in tropical and subtropical shallow-water marine habitats (Mathews, 2006). As burrowing crustaceans, snapping shrimps play ecologically critical roles in coastal ecosystems. They facilitate the material cycling of particulate matter through their foraging activities and perturbative behaviors. Burrowing shrimps alter the vertical profiles of sediment properties, thereby causing the redistribution of organic matter and nitrogen (Nacorda, 2008). Then, shrimps are omnivorous. Benthic microalgae and seagrass detritus are the primary dietary sources of snapping shrimps, accounting for 39% of their diet as reported in a previous study (Quigg et al., 2023). *Alpheus macellarius* harvested 0.02 and 0.06 g dry weight (DW) m^−2^ d^−1^ seagrass leaves in the dense and sparse meadows, respectively (Vonk et al., 2008). Significant insights have been gained into the behavior and ecological functions of snapping shrimps, but their intestinal microbiota and its relationship with the ambient environment remain to be in-depth explored. The seagrass sediment and dietary composition (e.g., seagrass detritus) may significantly influence the structure of intestinal microbiota in snapping shrimps.

In this study, we characterized the intestinal microbial composition of snapping shrimp *A. brevicristatus* collected from a seagrass bed, and compared it with the microbiota of surrounding sediment and seawater using 16S rRNA gene sequencing. This kind of snapping shrimp is widely distributed in the China seas, but has been rarely recorded or illustrated (Sha et al., 2019). This study provides fundamental information on the intestinal microbiota of this species in a natural environment, thereby paving the way for future studies on the microbiota’s potential functions in host environmental adaptation.

## 2 Materials and methods

### 2.1 Sample collection and preparation

Adult snapping shrimp *A. brevicristatus* including 6 males (MS) and 3 females (FS) were captured on 8 April, 2022 from a shallow seagrass bed in Beihai, Guangxi Province, China (21.4322° N, 109.2835° E). The specimens were transported to the lab in a tank at 4 °C with *in situ* seawater and immediately dissected to collect their intestines. Seawater samples (SW, *n* = 4) were collected in sterile polythene bags from the same locality. A 1-liter volume of seawater was passed through a 0.2 μm-pore-size hydrophilic polycarbonate membrane (Merck Millipore, Germany) to collect microorganisms. Sediment push-cores (Sed, *n* = 5) covering 0 to 50 cm depth were also taken using a ring knife. In total, 18 samples (9 intestines, 4 seawater filter membranes, and 5 surface sediments) were collected and preserved at −80 °C.

### 2.2 16S rRNA gene sequencing and data processing

Genomic DNA was extracted from an entire intestine or 0.25 g of sediment using the DNeasy PowerSoil Pro Kit (QIAGEN, Germany), and from a filter using the DNeasy PowerWater Kit (QIAGEN, Germany) according to the manufacturer’s protocol. The V3–V4 hypervariable regions of bacterial 16S rRNA genes were amplified by PCR with the universal primers 338F (5’-ACTCCTACGGGAGGCAGCA-3′) and 806R (5’-GGACTACHVGGGTWTCTAAT-3′) (Caporaso et al., 2011). PCR amplification was performed with a 50 μL mix containing 0.5 μL PrimeSTAR HS DNA Polymerase (Takara, Japan), 10 μL 5-fold reaction buffer (Mg^2+^ plus), 4 μL dNTP mixture (2.5 mM each), 2 μL of template DNA (~100 ng) or sterile water (blank control), 1 μL of each primer (10 μM) and 31.5 μL ddH_2_O. The PCR conditions were as follows: an initial denaturation at 98 °C for 30 s, 28 cycles of 98 °C denaturation for 10 s, 50 °C annealing for 30 s, 72 °C extension for 30 s, and a final extension at 72 °C for 5 min. The PCR products were detected by 1.2% agarose gel electrophoresis and subsequently purified using a MinElute GelExtraction Kit (QIAGEN, Germany). Three technical replicates were conducted for each PCR reaction, and the recovered DNA products were pooled. The Qubit 4.0 Fluorometer (Thermo Scientific, USA) was used to measure the concentration of PCR products. High-throughput sequencing was finished on the Illumina NovaSeq platform by Personal Biotechnology Co., Ltd. (Shanghai, China).

Paired-end reads (250 bp × 2) generated from the 18 samples were processed using the QIIME2 version 2024.5 platform (Bolyen et al., 2019). Raw reads were demultiplexed according to the unique oligonucleotide barcodes ligated to 5′ end of primers. The q2-cutadapt plugin was used to remove adapter, barcode, and primer sequences (Martin, 2011). Then, raw reads were quality filtered, denoised and merged. After removing chimeric sequences, unique amplicon sequence variants (ASVs) were generated using the q2-dada2 plugin (Callahan et al., 2016). Taxonomic classification was performed by Vsearch global alignment with default thresholds, using the SILVA database (release 132) as the reference (Rognes et al., 2016). ASVs classified as mitochondria, chloroplast and eukaryote were filtered out, as well as ASVs with frequency less than 5. The taxonomic profiles of samples were visualized using the q2-taxa plugin. Relative abundance of specific taxa was statistically measured by the Kruskal-Wallis test; and pairwise group comparison was performed by the Dunn’s post-hoc test with Bonferroni multiple-testing correction using the PAST v5.2.1 software (Hammer et al., 2001).

### 2.3 Alpha and beta diversity estimation

Sampling depth was normalized based on the minimum number of sequence (46,498) in the samples. Alpha diversity metrics, including community richness (Observed Features), evenness (Pielou’s Evenness) and diversity (Shannon Entropy and Faith’s Phylogenetic Diversity), were estimated using the q2-diversity plugin. Significant difference between groups was detected using the non-parametric Kruskal-Wallis test. When multiple comparisons were performed, a Benjamini-Hochberg false discovery rate (FDR) correction was performed and an adjusted *p* value (*q* value) < 0.05 was considered significant. Rarefaction curves were also calculated from the non-rarefied ASV table. To estimate beta diversity, both weighted and unweighted UniFrac distance matrixes were calculated from the rarefied ASV table using the q2-diversity plugin. Principal coordinates analysis (PCoA) plots were used to visualize ordinations using the OmicShare tools (Mu et al., 2024). A beta dispersion test (PERMDISP) was used to ascertain if observed differences were influenced by dispersion. ANOSIM (Analysis of Similarities) was used to determine significance in dissimilarity matrices across sample groups. Microbial biomarkers were identified using the LEfSe v1.1.2 software with a significance threshold of *p* < 0.05 and an LDA score cutoff >3.0 (Segata et al., 2011). Additionally, an UpSet diagram based on the presence or absence of ASVs was generated from the rarefied ASV table using the OmicShare tools.

### 2.4 Analysis of bacterial community assembly

Two distinct models were employed to compare the assembly mechanisms of shrimp intestinal and environmental microbiota (Zhou and Ning, 2017). First, using the “MicEco” package in R, stochastic processes involved in assembling bacterial communities were quantified through Neutral Community Model (NCM) fitting (Burns et al., 2016). The overall goodness of fit to the model (R-squared value) and the estimated migration rate of community (m value) were calculated at the same time. Second, a null model analysis was carried out with the R package “microeco” to evaluate the relative importance of determinism and stochasticity in microbiome assembly (Liu et al., 2021). The beta Nearest Taxon Index (*β*NTI) was calculated based on null model test of the phylogenetic β-diversity index βMNTD (β mean nearest-taxon distance). |βNTI| > 2 indicates the dominance of deterministic processes, which can be further divided into homogeneous selection (βNTI < −2) and heterogeneous selection (βNTI > 2). For pairwise comparisons with |βNTI| < 2, we further analyzed using the Bray-Curtis-based Raup-Crick (RC_bray_) metric, which quantifies the deviation between the observed Bray-Curtis and the null distribution. RC_bray_ > 0.95 signifies dispersal limitation, RC_bray_ < −0.95 denotes homogenizing dispersal, and |RC_bray_| ≤ 0.95 represents undominated processes (Stegen et al., 2013, 2015).

## 3 Results

### 3.1 High-throughput sequencing and data processing

A total of 5,185,250 paired-end reads were obtained from all intestinal and environmental samples, ranging from 198,897 to 407,006 reads per sample. After sequence processing, 1,681,695 high-quality sequences were retained and clustered into 9,014 ASVs. When aligned against the SILVA database, these ASVs were annotated to 69 phyla. The rarefaction curves based on observed features displayed reasonable degrees of saturation for all samples, so deeper sequencing could potentially uncover rare ASVs (Supplementary Figure S1).

### 3.2 Comparative analysis of microbial diversity

**Figure 1:**
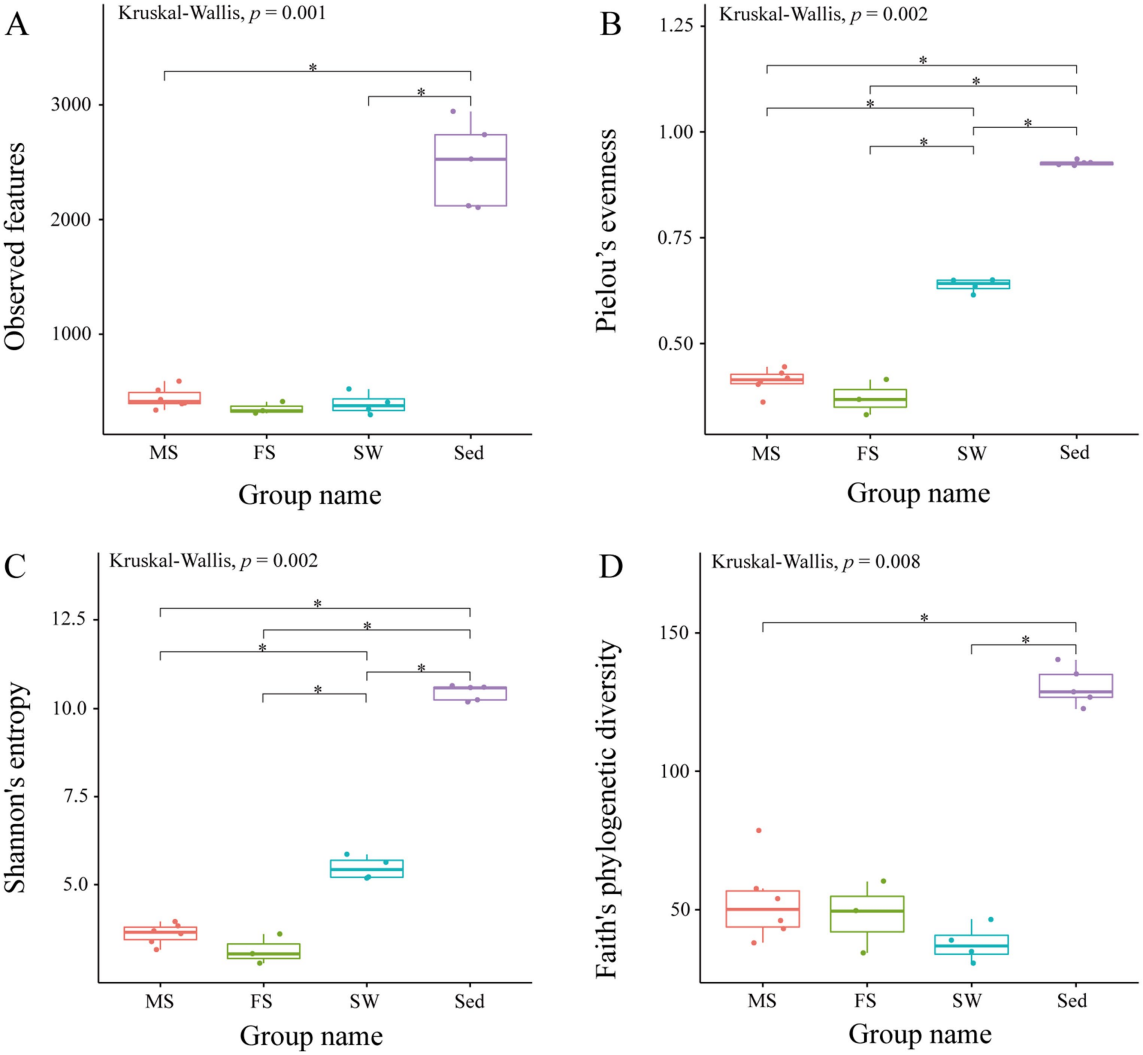
Alpha diversity comparison of microbiota among shrimp intestine, ambient seawater, and surface sediment. The indices, including **(A)** Observed features, **(B)** Pielou’s evenness, **(C)** Shannon entropy, and **(D)** Faith’s phylogenetic diversity, are used to describe alpha diversity. Alpha diversity is significantly higher in sediment than in shrimp intestines. The asterisk (*) denotes statistical significance between groups, with significance defined as a Benjamini-Hochberg *Q* value less than 0.05.

**Figure 2:**
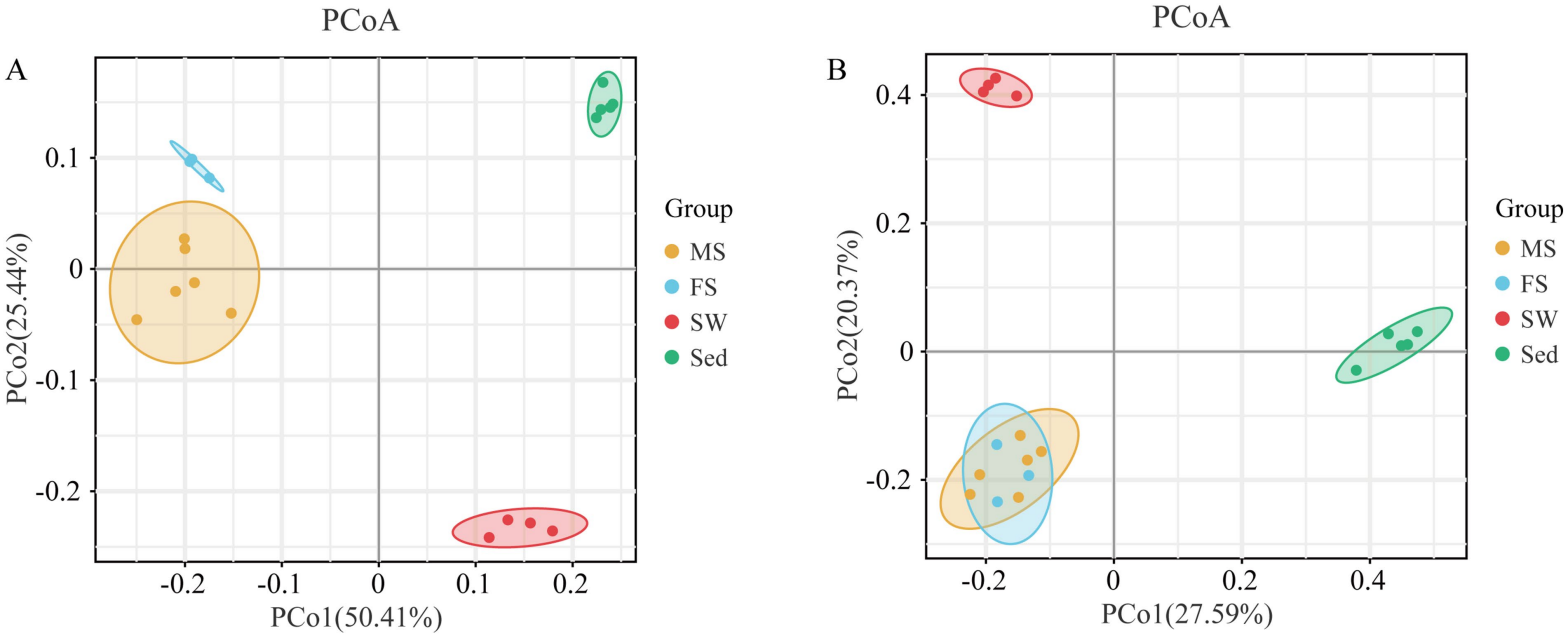
Beta diversity of bacterial communities across samples. Principal coordinates analysis (PCoA) is performed to compare bacterial communities across samples, based on **(A)** weighted and **(B)** unweighted UniFrac distances at the ASV level. The four sample groups were clearly separated from each other. Component axes indicate the percentage of variance explained.

Species richness, evenness, and diversity of the microbiota in the shrimp intestine, ambient seawater and surface sediment were compared (Figure 1). All examined alpha diversity indices demonstrated statistically significant differences among all groups (Kruskal-Wallis test, *p* < 0.01). Notably, the Observed Features and Faith’s Phylogenetic Diversity indices were markedly higher in the Sed group than in the MS and SW groups (pairwise Kruskal-Wallis test, *q* < 0.05), but did not differ significantly among the MS, FS and SW groups. Regarding community evenness and diversity, the Pielou’s Evenness and Shannon Entropy indices were highest in sediment microbial communities, followed by ambient seawater, and lowest in the snapping shrimp intestine (*q* < 0.05). Nevertheless, no significant difference was observed between the MS and FS groups. The similarities and differences in microbial communities between samples were evaluated by beta diversity analysis. The PCoA result based on weighted UniFrac distance showed that the four sample groups were clearly separated from each other. In other words, intragroup differences were less than intergroup differences (ANOSIM, *q* < 0.05) (Figure 2A; Supplementary Table S1). However, the FS and MS groups were clustered together in the reduced-dimension map based on unweighted UniFrac distance, and ANOSIM revealed no significant differences between these two groups (*q* > 0.05) (Figure 2B; Supplementary Table S1).

### 3.3 Taxonomic composition of intestinal and environmental microbiota

The bacterial compositions within the shrimp intestine were compared with those present in its surrounding environment. Results showed that the intestinal microbiota of *A. brevicristatus* were primarily dominated by Proteobacteria (mean ± SD, FS: 78.11 ± 9.48%, MS: 68.70 ± 10.84%) and Firmicutes (FS: 16.71 ± 10.33%, MS: 27.53 ± 9.86%), which collectively accounted for over 92% of the microbial communities (Supplementary Figure S2). Proteobacteria (63.47 ± 6.97%) was also the most abundant phylum in the SW group, followed by Actinobacteriota (17.20 ± 3.49%) and Bacteroidota (16.00 ± 4.15%). Within the Sed group, the top 3 dominant phyla were Proteobacteria (20.13 ± 2.88%), Desulfobacterota (16.82 ± 1.32%) and Bacteroidota (16.76 ± 1.22%).

**Figure 3:**
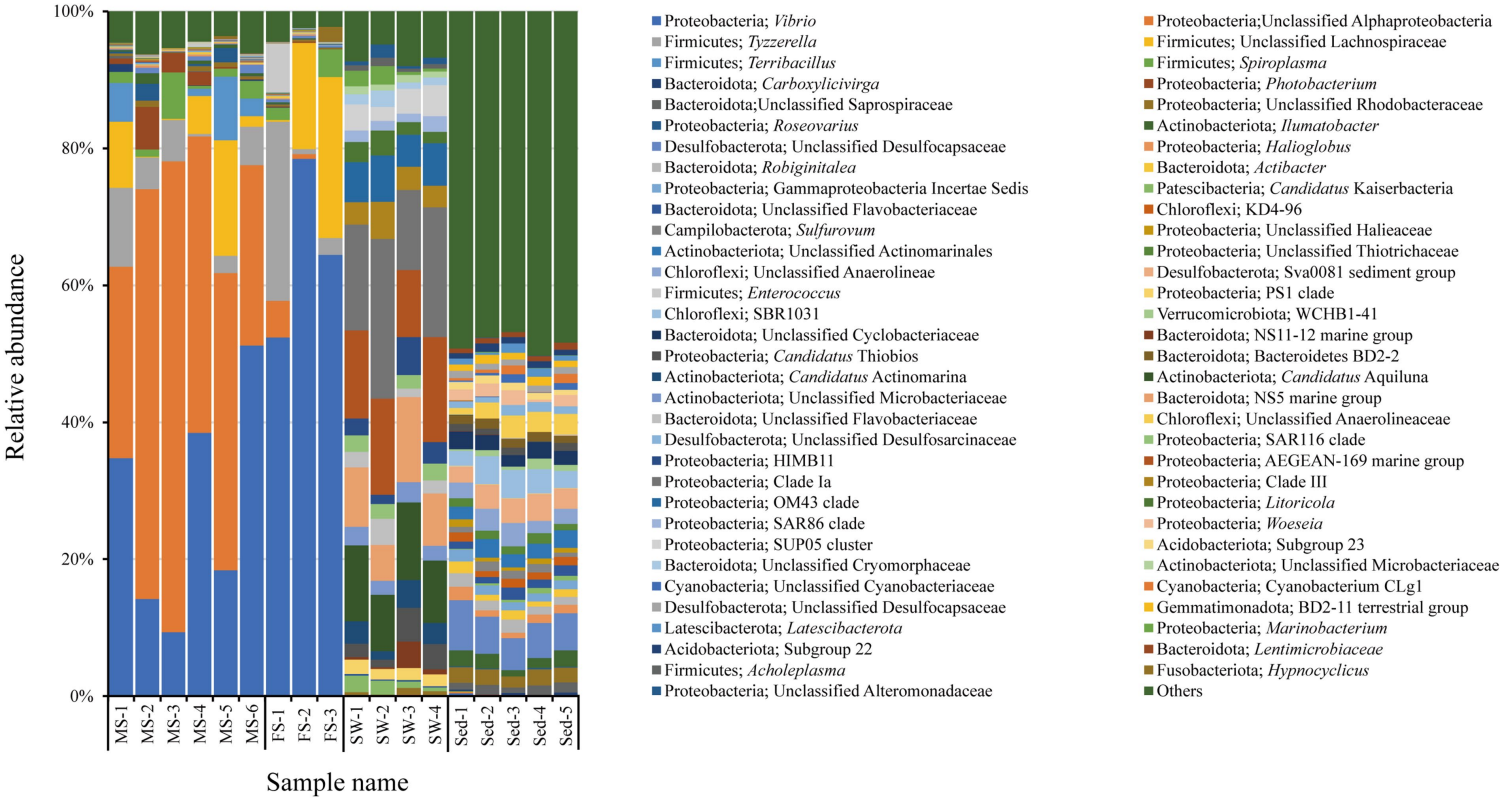
Microbial community composition in shrimp intestine, ambient seawater, and surface sediment displayed at the genus level. Genus with percentage less than 1% in all samples is classified into others. The intestinal microbiota were dominated by unclassified Alphaproteobacteria and *Vibrio*.

At the genus level, the intestinal microbiota of the MS group was predominantly composed of unclassified Alphaproteobacteria (44.91 ± 16.90%) and *Vibrio* (27.75 ± 16.27%) (Figure 3); Moreover, the relative abundance of unclassified Alphaproteobacteria was significantly higher in the MS group compared to the SW (0.05 ± 0.03%) and Sed (0.06 ± 0.05%) groups (Kruskal-Wallis test with Bonferroni-Dunn’s *post hoc* test, *q* < 0.05). *Vibrio* was also the most abundant genus in the FS group (65.00 ± 13.08%), significantly higher than in both the SW and Sed groups (SW, 0.07 ± 0.04%; Sed, 0.11 ± 0.18%, *q* < 0.05). The ASVs of unclassified Alphaproteobacteria and *Vibrio* with the highest frequencies were extracted. When searched against the NCBI 16S ribosomal RNA sequence database, these ASVs had the highest identity with *Agrobacterium fabrum* strain C58 (NR 074266, 86.73% identity) and *Vibrio jasicida* CAIM 1864 (NR 113182, 100% identity), respectively. The top 3 dominant genera in the SW group were Clade Ia (17.36 ± 4.97%), AEGEAN-169 marine group (13.01 ± 2.35%) and *Candidatus* Aquiluna (9.94 ± 1.50%). While in the Sed group, the dominant genus was unclassified Desulfocapsaceae (5.57 ± 1.01%), followed by SBR1031 (3.31 ± 0.91%) and Sva0081 sediment group (3.28 ± 0.61%).

### 3.4 Featured microbes in shrimp’s intestine and environment

Based on the ASV cluster analysis, the UpSet diagram revealed that the MS, FS, Sed, and SW groups had 1,173, 388, 5,821, and 774 unique ASVs, respectively (Supplementary Figure S3). Notably, only 11 ASVs were found to be common across all four groups, highlighting the limited overlap in microbial composition. Furthermore, 564 ASVs were shared between the shrimp intestines (MS and FS groups) and surrounding sediment, whereas merely 28 ASVs overlapped between the intestines and seawater.

**Figure 4:**
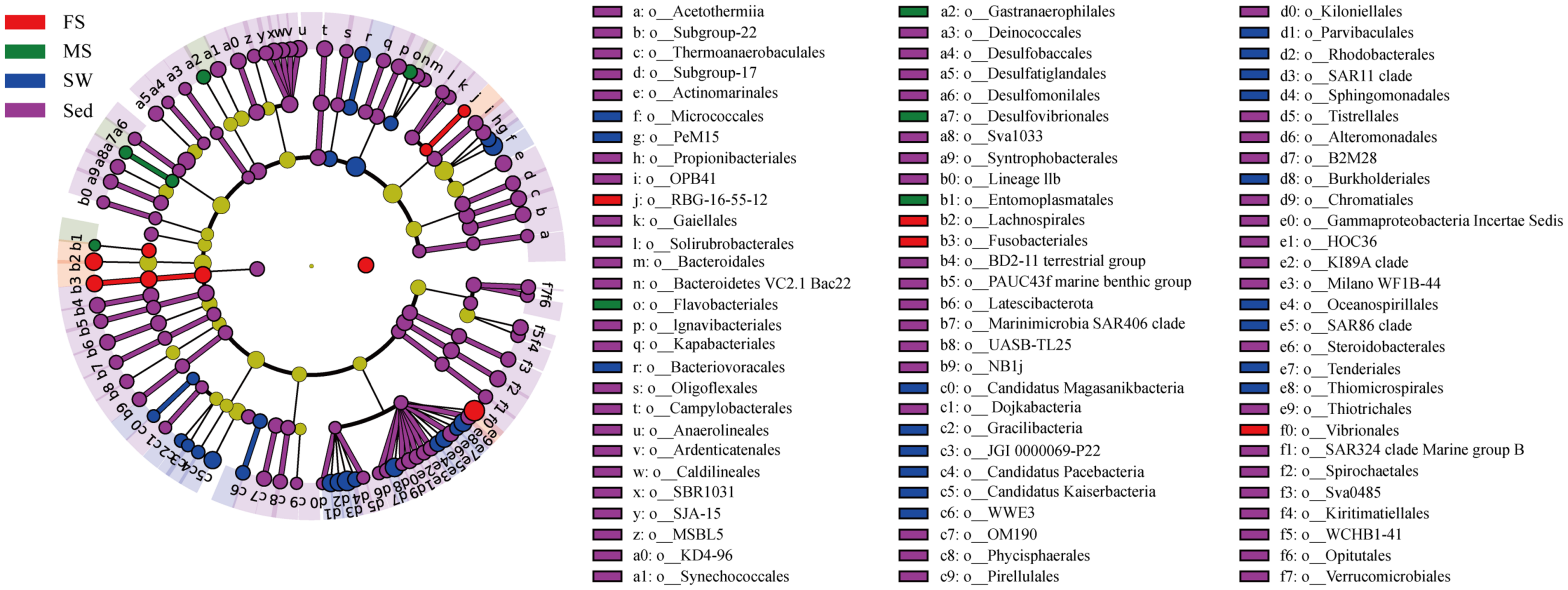
LEfSe analysis of differentially abundant microbial clades (Kruskal-Wallis test, *p* < 0.05; LDA score > 3.0). Only discriminative taxa in order level were displayed. Circles radiating from inside to outside represent taxonomic levels from phylum to order. The root of the cladogram denotes the domain Bacteria. Bacterial clades with significant differences are colored by group.

Furthermore, 84 discriminative taxa in order level (LDA score >3) among four groups were identified by LEfSe method. The cladogram illustrating the phylogenetic relationships of these significantly different taxa (Figure 4). In the FS group, four taxa were significantly enriched, including the orders RBG-16-55-12, Lachnospirales, Fusobacteriales, and Vibrionales. In contrast, Flavobacteriales, Gastranaerophilales, Desulfovibrionales, and Entomoplasmatales were significantly enriched in the MS group. Apart from that, 18 and 58 discriminative taxa were significantly enriched in the SW and Sed groups, respectively.

### 3.5 Quantify the process of community construction

**Figure 5:**
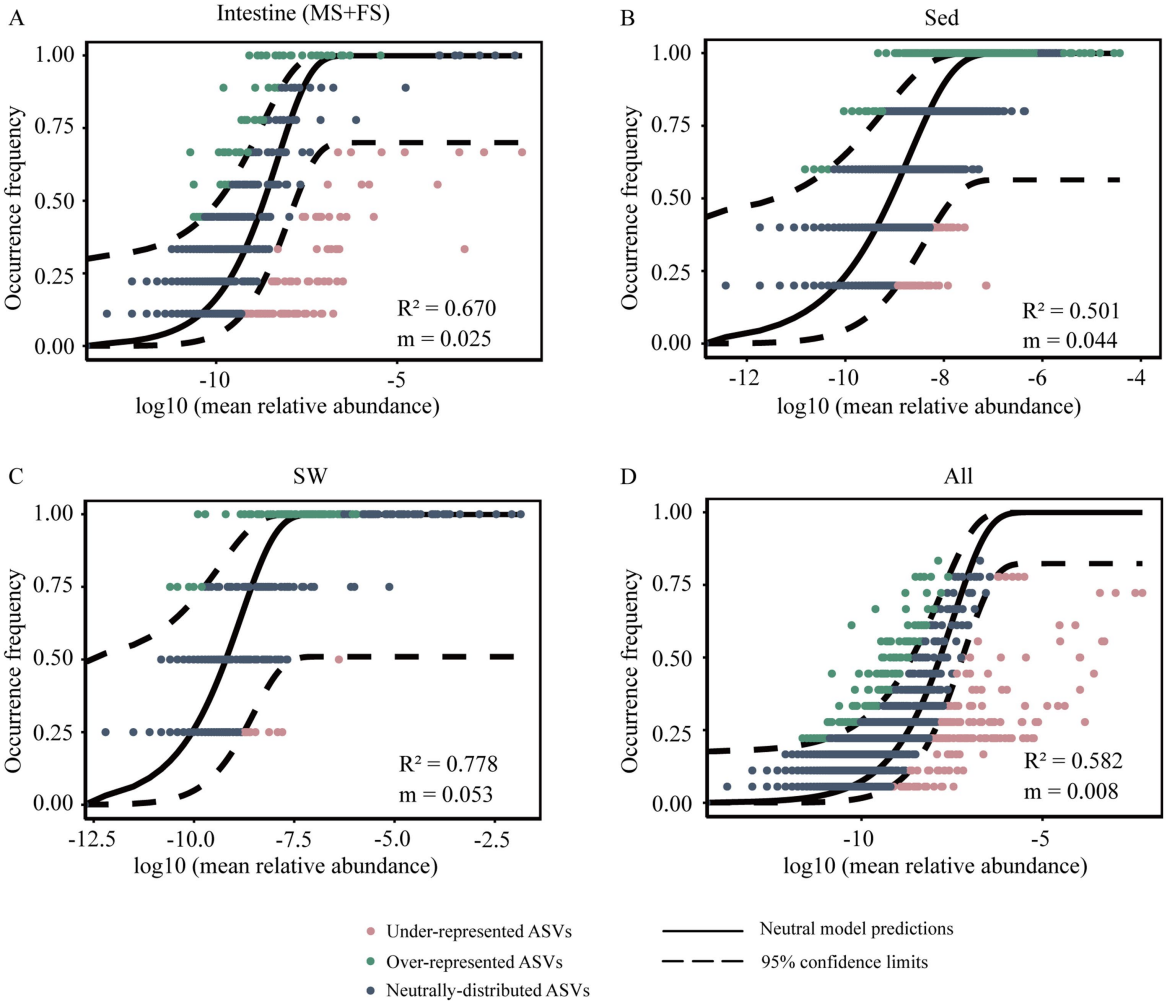
Fit of the neutral community model (NCM) of bacterial community assembly in shrimp intestine (**A**), surface sediment (**B**), ambient seawater (**C**), and all samples (**D**). ASVs that occur more frequently (blackish green) or less frequently (red) than predicted (blue) by the NCM are shown in different colors. The solid blue lines indicate the best fit to the NCM. The dashed lines indicate the 95% confidence interval around the neutral model. *R*^2^: The overall goodness of fit to the model; *m*: the estimated migration rate of community.

**Figure 6:**
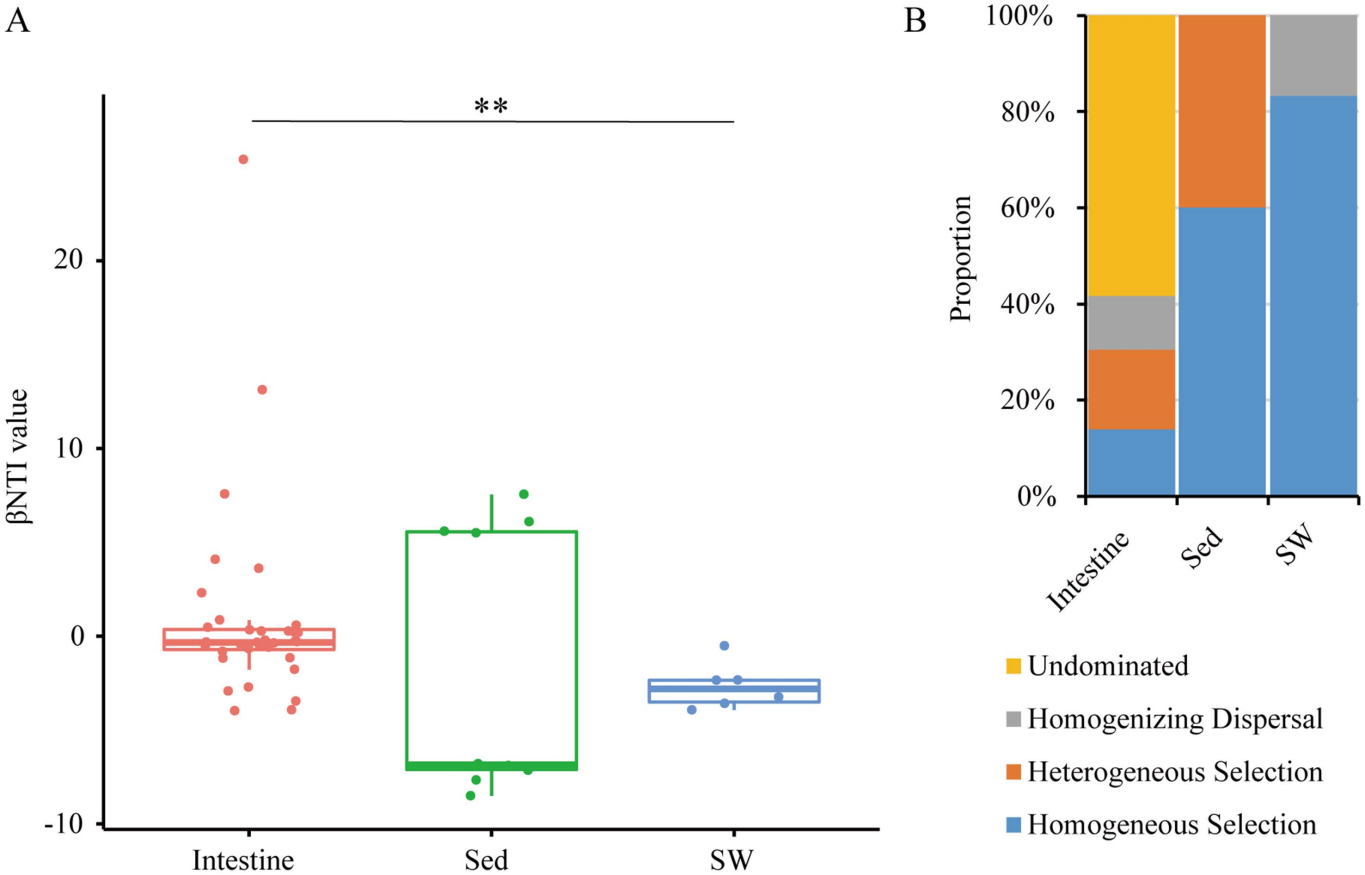
Mechanisms of bacterial community assembly in shrimp intestine, sediment, and seawater as evaluated by null model analysis. Distribution of beta nearest taxon index (*β*-NTI) are calculated across different samples **(A)**, and the proportion of dispersal limitation, homogenizing dispersal, and undominated process in the microbial assembly process are also estimated **(B)**.

The neutral model exhibited a goodness-of-fit of 0.670 when applied to the shrimp intestinal microbiome (MS and FS groups). This value was higher than that observed in sediment (*R*^2^ = 0.501), but lower than that in surrounding seawater (*R*^2^ = 0.778) (Figure 5), which potentially reflected the relative importance of stochastic processes versus deterministic processes across different environments. The higher the *R*^2^ value, the more closely the community aligns with the neutral mode, that is, stochastic processes have a greater influence on community construction. Moreover, the neutral model estimated a lower m value (migration rate, 0.025) in the intestinal bacterial community, implying the extremely low capability of microbes to disperse among and from intestines. Further, through null model analysis, the combination of βNTI and RC_bray_ was employed to approximate the relative contributions of different processes in community assembly. The βNTI values were used to assess the different ecological assembly processes in microbial communities (Figure 6A). For intestinal bacterial communities of snapping shrimps, undominated process was the most important ecological process and explained 58.3% of community turnover (Figure 6B). In addition, homogenizing dispersal, homogeneous selection and heterogeneous selection accounted for 11.1, 13.9, and 16.7%, respectively. Bacterial communities of sediments were governed by deterministic processes, including homogeneous selection (60.0%) and heterogeneous selection (40.0%). The community assemblies in seawater were mainly influenced by homogeneous selection (83.3%), followed by homogenizing dispersal (16.7%).

## 4 Discussion

In this study, the intestinal microbiota of *Alpheus brevicristatus* sampled from a seagrass bed was investigated, with additional comparisons made to the habitat-associated microbiota in both sediment and seawater. Earlier studies have explored the effects of shrimp behavior on seagrass ecosystems (Nacorda, 2008; Vonk et al., 2008), but they have neglected the role of microorganisms in the interaction between benthos and seagrass bed. According to the results of high-throughput sequencing, Proteobacteria was the most abundant bacterial phylum in the intestine of *A. brevicristatus*. Moreover, at the genus level, the intestines were predominantly occupied by unclassified Alphaproteobacteria and *Vibrio*. At the phylum level, the intestinal microbiota of *A. brevicristatus* showed a relatively high consistency with other decapod shrimps, including the commercially important *Litopenaeus vannamei* and *Macrobrachium nipponense* (Zeng et al., 2017; Zhao et al., 2018; Tang et al., 2021). It was reported that Proteobacteria were quite stable in the intestine of *L. vannamei*, and their abundance did not vary with ambient salinity, sulfide exposure and diet composition (Li et al., 2018). The dominance of Proteobacteria in the intestinal microbiota may be largely attributed to their adaptability to the intestinal microenvironment. Members of this phylum exhibit considerable morphological diversity and possess highly versatile physiological capabilities (Shin et al., 2015). However, the composition of the intestinal microbial community at the genus level was not entirely the same. The top 3 genera in the intestine of *L. vannamei* were *Candidatus* Xiphinematobacter, *Propionigenium*, and *Synechococcus*, which accounted for 3.4, 3.4, and 2.7%, respectively (Zeng et al., 2017). While, in the intestine of adult *Penaeus monodon*, *Actinotalea* accounted for more than 60% (Cicala et al., 2020). The differences at the genus level may result from the combined effects of environment, diet, and breeding conditions. Meanwhile, the intestinal microbiota in shrimps maintains functional stability (at the phylum level) while being able to adapt to environmental changes (at the genus level). The unclassified Alphaproteobacteria seems to be a novel taxonomic group, because its representative 16S amplicon sequence shared less than 90% identity with the top hit (*Agrobacterium fabrum*) in the database. Its function in the intestinal ecosystem of snapping shrimp, as well as that of *Vibrio*, requires further studies.

The alpha index analysis demonstrated that the sediment exhibited significantly higher diversity than the shrimp intestine and seawater. Then, beta diversity analysis demonstrated clear distinctions in microbial community composition across different sample types. PCoA based on weighted UniFrac distances showed a significant separation between all four groups. The lower alpha diversity in the shrimp intestine may be due to the selective pressure exerted by the host. The shrimp intestine possesses an effective innate immune system that can control bacterial proliferation by producing a series of antimicrobial peptides and reactive oxygen species (Zhou et al., 2025). So, intestine environment of shrimp is a strong habitat filter for the microbial community (Zhang and Sun, 2022). Moreover, the structure of the intestinal microbial community was strongly shaped by habitat type (El-Saadony et al., 2022). Key environmental factors, such as organic matter content, salinity, redox potential, and oxygen level, differ dramatically among the shrimp intestine, sediment, and seawater. These disparities drive each habitat to foster distinct microbial assemblages. Study suggests that the composition and characteristics of shrimp intestinal microbiota are predominantly influenced by the aquatic environment (Zhou et al., 2025). More than 77% of the intestinal microbes of *L. vannamei* from a shrimp-culturing pond were represented in either the sediment or the surrounding water (Hou et al., 2018). But, in this study, a notably limited overlap of ASVs across different sample groups were revealed, and only 11 ASVs were found to be shared by all groups. Furthermore, the shrimp intestine shared 564 ASVs with sediment, compared to only 28 with seawater. A similar situation was observed in *Penaeus japonicus*; sediment served as a more substantial reservoir and source of intestinal microbes when compared to seawater (Zhou et al., 2021). The conflicting research findings may be associated with the host’s diet and development (Huang et al., 2018).

The seawater microbial communities exhibited a high NCM goodness of fit (*R*^2^ = 0.778), indicating that stochastic processes dominated in shaping microbial community diversity. The finding is consistent with previous studies (Yu et al., 2023; Zheng et al., 2024). Most microorganisms have a greater dispersal ability than macroorganisms because their tiny size allows them to be transported easily by water flow. Meanwhile, seawater environments are highly homogeneous (e.g., in temperature and salinity), resulting in weak niche differentiation. However, marine sediment serves as a complex microhabitat with heterogeneous microenvironments (Mallik et al., 2024). Microorganisms must adapt to distinct microenvironment, so environmental filtering becomes the primary driver of community assembly (Petro et al., 2017). The *R*^2^ value for intestinal microbiota (0.670) was lower than that for seawater but higher than for sediment, indicating that stochastic processes still worked appreciably, yet deterministic forces began to shape the community. The null model analysis further validated that stochastic processes, including undominated processes and homogenizing dispersal, had a weight of 69.4% in intestinal microbial community assembly. Initially, the host filters out most ingested microbes via physical barriers, chemical factors, and immune responses; and only a small subset of “tolerant” microorganisms survived. After this, stochastic processes may become the primary drivers of community turnover. Host filtering may sets the boundary of “which microbes are allowed to enter the intestine,” whereas stochastic processes govern the assembly dynamics of “how these survived microbes fluctuate within the intestine.” Even more, the shrimp intestine is structurally simpler than that of many other aquatic animals, such as fish. The intestine is short and straight, and lacks the tortuous folding structures (Zhou et al., 2025). Ingested material resides in the shrimp intestine for only a brief period, which may enhance the passive dispersal of transient microbe. The extremely low migration rate (m = 0.025) for the shrimp intestine indicated that microbial dispersal between individual hosts was quite limited. These findings highlight the complexity of microbial community assembly in *A. brevicristatus* intestine, as a host-associated microhabitat, represents a balance between ecological drift and host-mediated selection. Understanding these assembly mechanisms is critical for predicting how microbial communities respond to environmental changes and, in turn, their impacts on host health and ecosystem function.

This study provides a foundational understanding of the intestinal and environmental microbiota associated with *A. brevicristatus*, but several limitations should be noted. First, the sample size is relatively small, which may limit the generalizability of the results. Future studies should increase the sample size. Multiple sampling sites and seasons should be included to account for spatial and temporal variations. Second, this study only focuses on taxonomic composition rather than function, and metagenomic and metatranscriptomic analyses can provide insights into the functional roles of microbial communities and their interactions with the host.

## 5 Conclusion

This study presents the first comparative analysis of the intestinal and environmental microbiota of the seagrass bed-dwelling snapping shrimp *Alpheus brevicristatus*. We demonstrate that the intestines of *A. brevicristatus* harbor unique microbial communities, dominated by unclassified Alphaproteobacteria and *Vibrio*. Despite living in close contact with sediment and seawater, their intestinal microbial communities share only a small proportion of taxa with surrounding environment, indicating strong host filtering. Notably, microbial community assembly in the snapping shrimp intestine is governed by both stochastic and deterministic processes. This suggests that internal dynamics and random colonization events play a critical role as well as host filtering in shaping the intestinal microbiome of this invertebrate. The distinct microbial communities of *A. brevicristatus* and its environment, together with the unique assembly mechanisms identified in this study, provide a framework for future research on the ecological and evolutionary significance of intestinal microbiota in snapping shrimp and other benthic invertebrates.

## Data Availability

The datasets presented in this study can be found in online repositories. The names of the repository/repositories and accession number(s) can be found at: https://db.cngb.org/cnsa/, CNP0006743.

## Supplementary material

SUPPLEMENTARY FIGURE S1 Alpha rarefaction curves of observed features for all samples.

SUPPLEMENTARY FIGURE S2 Microbial community composition in the shrimp intestine, ambient seawater and surface sediment displayed at the phylum level. Phylum with percentage less than 1% in all samples is classified into others.

SUPPLEMENTARY FIGURE S3 Upset plot of ASV co-occurrence patterns across shrimp intestine, ambient seawater and surface sediment.

SUPPLEMENTARY TABLE S1 Pairwise ANOSIM for beta diversity analysis.
