# Supplementary figures and images for: Comparison of intestinal and environmental microbiota of the snapping shrimp (Alpheus brevicristatus) in a seagrass bed
Source: Front Microbiol. 2026 Jan 9;16:1735708. doi: 10.3389/fmicb.2025.1735708 (PMC12827752; doi:10.3389/fmicb.2025.1735708)

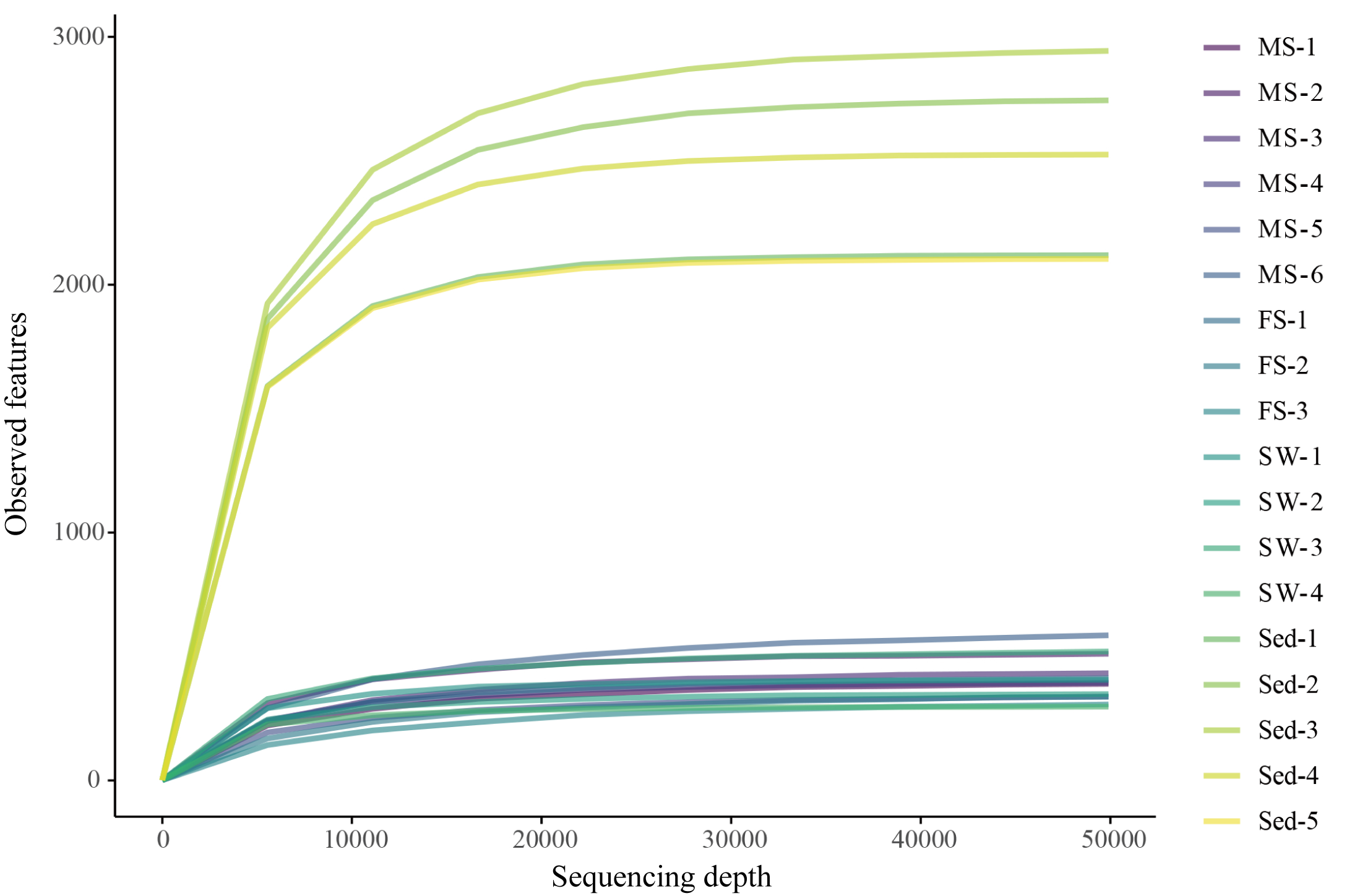

Supplement: SUPPLEMENTARY FIGURE S1 — Alpha rarefaction curves of observed features for all samples. [file Image_1.TIF]

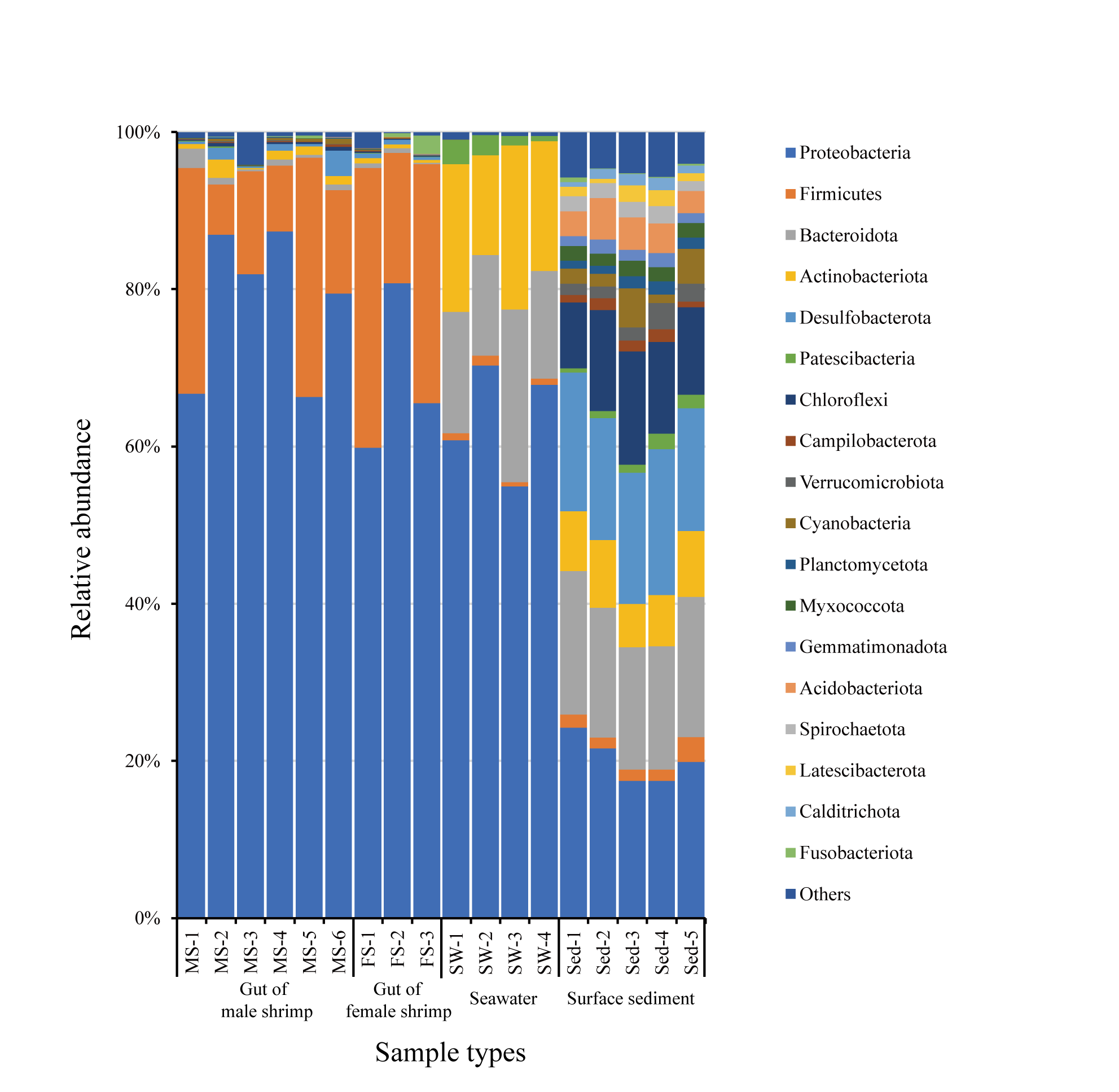

Supplement: SUPPLEMENTARY FIGURE S2 — Microbial community composition in the shrimp intestine, ambient seawater and surface sediment displayed at the phylum level. Phylum with percentage less than 1% in all samples is classified into others. [file Image_2.TIF]

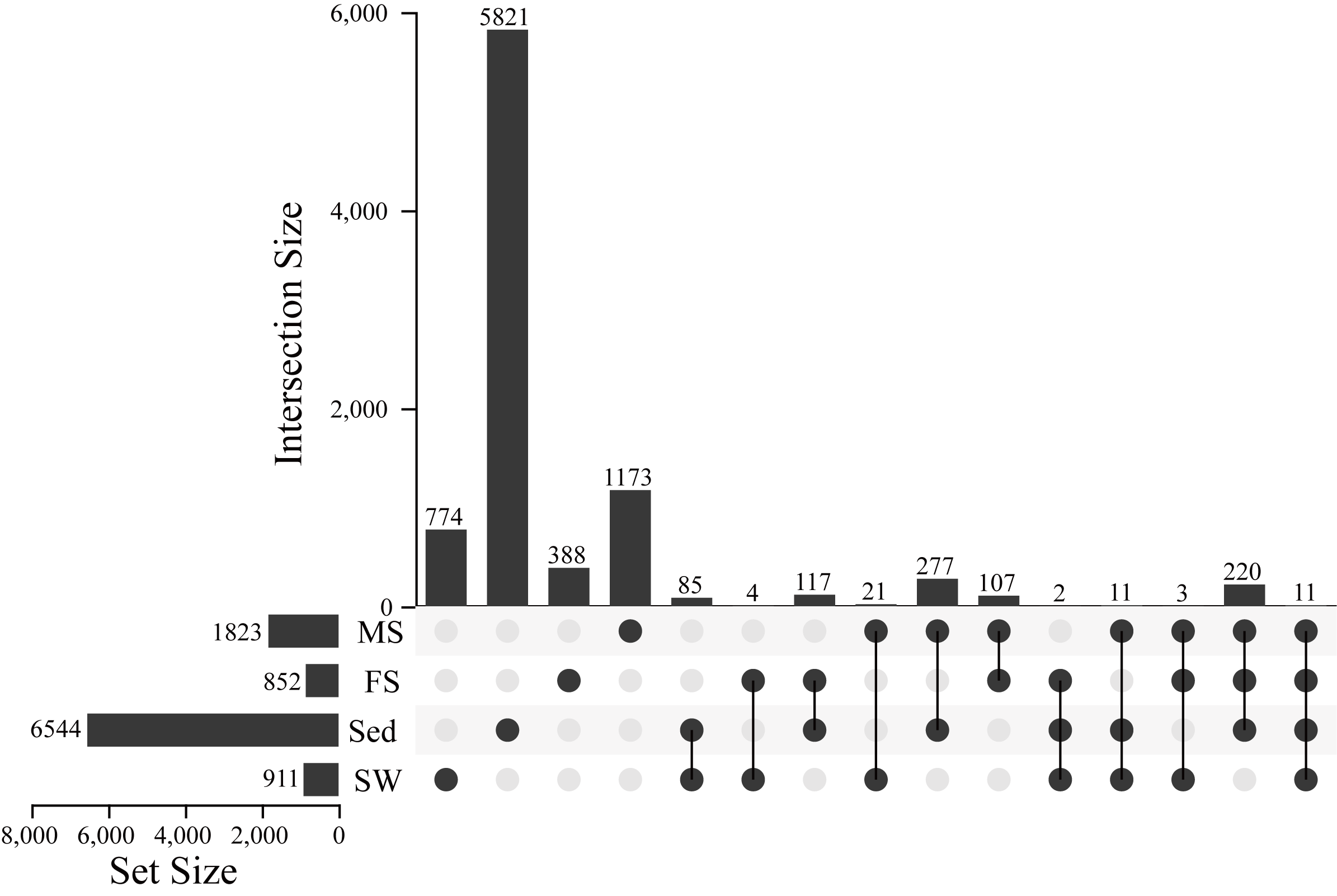

Supplement: SUPPLEMENTARY FIGURE S3 — Upset plot of ASV co-occurrence patterns across shrimp intestine, ambient seawater and surface sediment. [file Image_3.TIF]
