# Supplementary material for: Comparison of intestinal and environmental microbiota of the snapping shrimp (Alpheus brevicristatus) in a seagrass bed
Source: Front Microbiol. 2026 Jan 9;16:1735708. doi: 10.3389/fmicb.2025.1735708 (PMC12827752; doi:10.3389/fmicb.2025.1735708)
Supplement: SUPPLEMENTARY TABLE S1 — Pairwise ANOSIM for beta diversity analysis. [file Table_1.docx]

Table S1. Pairwise ANOSIM for beta diversity analysis.

| Group | R value | *p* value | BH adjusted *p* value (*q* value) |
| --- | --- | --- | --- |
| MS-vs-FS | 0.5123 | 0.0245 | 0.1470 |
| MS-vs-SW | 0.5079 | 0.0151 | 0.0906 |
| MS-vs-Sed | 0.5600 | 0.0071 | 0.0426 |
| FS-vs-SW | 1 | 0.0294 | 0.1764 |
| FS-vs-Sed | 1 | 0.0183 | 0.1098 |
| SW-vs-Sed | 1 | 0.0078 | 0.0468 |

Abbreviations: MS=male shrimp, FS=female shrimp, SW=seawater, Sed=sediment. R values represent difference of mean ranks between the two groups. Values closer to 1.0 indicate greater dissimilarity between the two groups compared.
